# Type-II band-engineered infrared detectors: III–V T2SLs, 2D junctions, and mixed-dimensional hybrids

**DOI:** 10.1039/d6ra05584b

**Published:** 2026-08-27

**Authors:** Yan Zhang, Wei Su, Wenhong Zhou

**Affiliations:** a Wuhan Guide Infrared Co., Ltd Wuhan 430000 Hubei China 13971650762@163.com; b Wuhan Global Sensor Tech. Ltd Co Wuhan 430000 Hubei China

## Abstract

Type-II band engineering spans several physically distinct infrared-detector platforms: periodic epitaxial III–V type-II superlattices (T2SLs), atomically layered 2D/2D type-II junctions, and mixed-dimensional structures in which a 2D layer serves as a barrier, contact, gate, or sensitizer for a T2SL or another absorber. This review separates these categories rather than treating them as interchangeable. We examine interface bonding and passivation, carrier transfer and gain, and optical or barrier-based enhancement across SWIR, MWIR, and LWIR detection. True T2SL/2D demonstrations remain limited, but matched-control studies show that MoS_2_ barriers can reduce T2SL dark current by about two orders of magnitude and that graphene coupling can enhance LWIR detectivity by 217-fold under the conditions of a single study. Reported figures of merit are compared only with their wavelength, temperature, bandwidth, bias, device area, and noise-extraction context; cross-study values are not treated as a rank order. We also place these platforms beside colloidal-quantum-dot, QWIP/QDIP, and nBn technologies, explain the gap between idealized calculations and measured devices, and assess chemical interface engineering, scalability, reliability, and commercial readiness.

## Abstract 2

This review illustrates how type-II superlattices, hybrid junctions, and 2D van der Waals heterostructures leverage interfacial charge transfer to regulate carrier transport and enhance SWIR-to-LWIR infrared detection.

## Graphical abstract

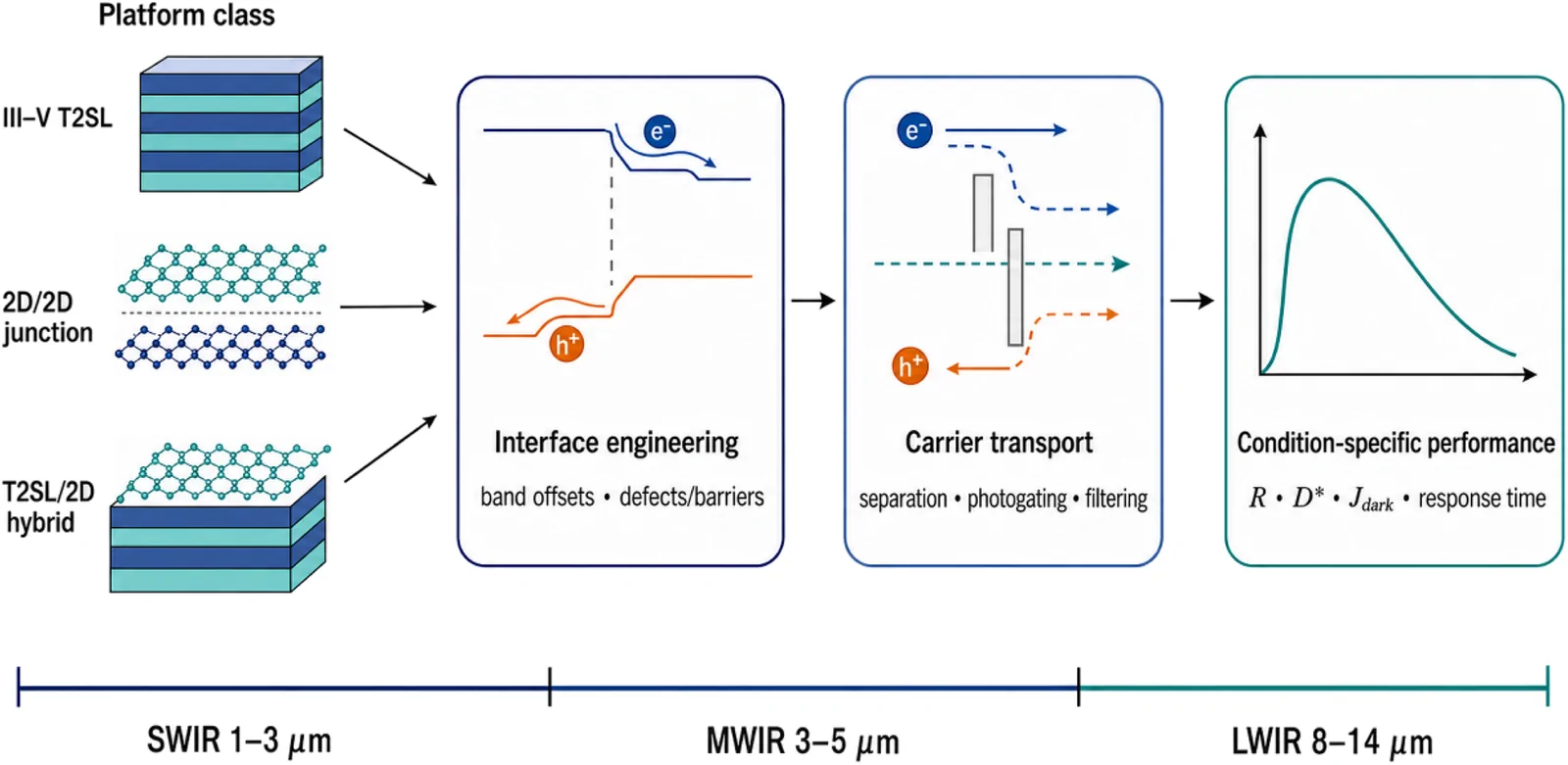

## 1. Introduction

Infrared photodetectors underpin surveillance, industrial monitoring, environmental sensing, medical instrumentation, and optical communication. Established HgCdTe, III–V superlattice, quantum-well, barrier-detector, and thermal-detector technologies occupy different wavelength, temperature, speed, uniformity, and cost regimes; therefore, no single platform provides a universal benchmark. The demand for smaller and lower-power systems has intensified research on band-engineered heterostructures that can suppress thermally generated current, enhance absorption, or add internal gain without obscuring the associated noise and speed penalties.^1–4^

The terminology requires a strict boundary. A type-II band alignment is an energetic arrangement in which the conduction-band minimum and valence-band maximum reside in different constituents. By contrast, a type-II superlattice denotes a periodic epitaxial quantum structure whose alternating narrow layers form electron and hole minibands, as in InAs/GaSb or InAs/InGaSb structures.^4,5^ A MoS_2_/WSe_2_ junction may therefore have type-II alignment without being a conventional III–V T2SL. This review uses “T2SL/2D hybrid” only when a 2D material is physically integrated with an epitaxial T2SL; 2D/2D and other mixed-dimensional type-II junctions are discussed as related but separate classes.

Three platform families are compared. Epitaxial T2SLs offer mature miniband design, long-wavelength absorption, and compatibility with focal-plane processing, but generally require demanding epitaxy and cooling for the lowest-noise LWIR operation. 2D/2D van der Waals junctions relax lattice-matching constraints and support gate-tunable bands, ultrathin vertical transport, and flexible spectral design, yet their small absorption volume, contact variability, trapping, and device-to-device dispersion remain serious limitations. True T2SL/2D hybrids can add a selective barrier, transparent contact, photogate, or tunable optical boundary to a T2SL absorber; their principal liabilities are transfer contamination, interface reproducibility, added process steps, and the small number of matched-control and array-level studies.^4,6–8^

**Fig. 1:**
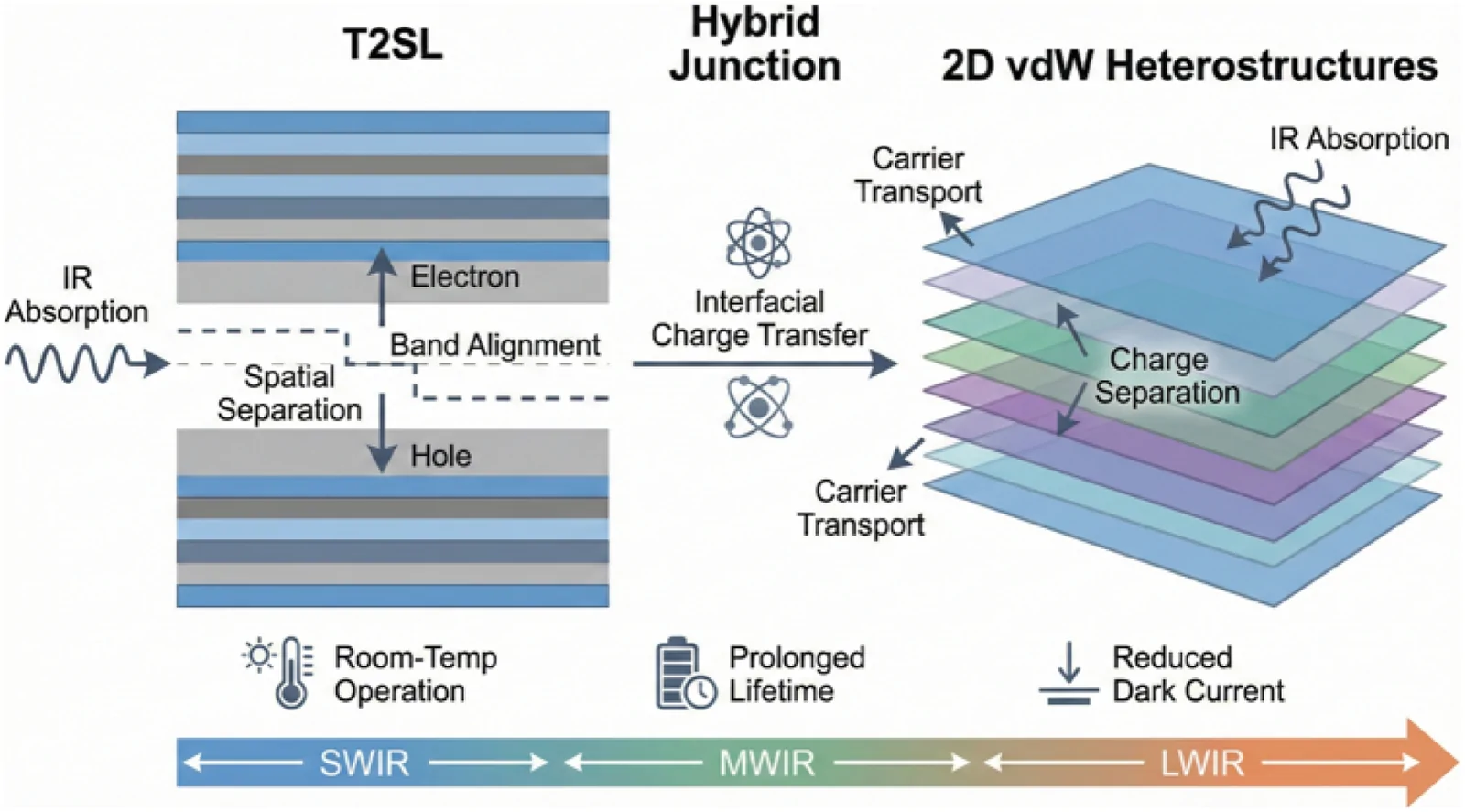
Scope and causal framework connecting platform class, interface chemistry, carrier transport, and condition-specific infrared-detector performance.

Fig. 1 defines the review framework. The discussion first distinguishes epitaxial T2SLs, 2D/2D type-II junctions, and true mixed-dimensional T2SL/2D hybrids; it then connects interface chemistry to carrier transport and finally to condition-specific device metrics. The scope covers SWIR (1–3 µm), MWIR (3–5 µm), and LWIR (8–14 µm) detection. The central questions are where a hybrid provides an experimentally demonstrated advantage over an appropriate constituent control, which gain or filtering mechanism produces that advantage, and whether the comparison remains valid after temperature, wavelength, bias, area, and noise bandwidth are considered. Because the genuine T2SL/2D literature is still small, the broader 2D type-II literature is used to identify transferable interface and transport principles, not as evidence that every type-II 2D junction is a T2SL.

### 1.1 Figures of merit and comparison rules

Responsivity *R* is electrical output per incident optical power (A W^−1^ for current readout). The external quantum efficiency is EQE = (*Rhc*)/(*qλ*) for a current-output detector; EQE above 100% denotes internal photoconductive or photogating gain and should be accompanied by bandwidth, linearity, and noise. Noise-equivalent power is NEP = *i*_n_/*R* in W Hz^−1/2^, where *i*_n_ is the measured current-noise spectral density. Specific detectivity normalizes for area A and bandwidth Δ*f*: D* = (*A*Δ*f*)^1/2^/NEP = *R*(*A*Δ*f*)^1/2^/*i*_n_, expressed in Jones (cm Hz^1/2^ W^−1^).^9^

Background-limited infrared photodetection (BLIP) identifies the regime in which photon-background fluctuations dominate other noise sources; it is not a universal D* value. For imaging arrays, noise-equivalent temperature difference (NETD) is the scene-temperature change that yields unit signal-to-noise ratio and depends on optics, integration time, readout, operability, and calibration. Every quantitative comparison in this review therefore retains wavelength, temperature, bias, active area, bandwidth or transient protocol, and the noise model. Measured noise is preferred to shot-noise-only estimates.^9^

## 2. Material systems and interface chemistry

### 2.1 Overview of type-II heterostructure platforms

The reviewed systems fall into distinct structural classes rather than one continuous T2SL/2D category (Table 1). Periodic III–V T2SLs generate minibands through epitaxial repetition; 2D/2D junctions rely on van der Waals stacking and interlayer band offsets; true T2SL/2D hybrids place a 2D layer on an epitaxial superlattice as a barrier, photogate, electrode, or optically active boundary. Other mixed-dimensional structures, including 2D/quantum-dot and 2D/bulk junctions, provide mechanistic analogies but must not be counted as T2SL/2D integrations.

**Table 1:** Physical scope of the type-II platforms covered in this review and the inferences that may be drawn from each class

Platform class	Physical definition	Representative systems	Interpretive boundary
Epitaxial T2SL	Periodic III–V quantum layers forming electron/hole minibands	InAs/GaSb; InAs/InGaSb^4,5^	Mature long-wave platform; not a 2D heterostructure
True T2SL/2D hybrid	2D layer integrated with an epitaxial T2SL absorber	MoS_2_/T2SL barrier; graphene/InAs–GaInSb T2SL^6,8^	Hybrid advantage requires a matched T2SL control
2D/2D type-II junction	van der Waals or lateral junction with staggered band edges	MoS_2_/WSe_2_; WSe_2_/WS_2_; InSe/PdSe_2_ (ref. 10–12)	Type-II alignment does not make the structure a conventional T2SL
Other mixed-dimensional	2D layer coupled to quantum dots or a bulk/oxide semiconductor	MoS_2_/PbS QDs; InAs@ZnSe/MoS_2_; MoS_2_/CdTe^13–15^	Mechanistic analogue; not T2SL/2D integration
Competing detector	Alternative absorber or barrier architecture	CQD; QWIP/QDIP; nBn^16–21^	Compare system metrics, not D* alone

InAs/GaSb and InAs/InGaSb T2SLs are the most mature long-wavelength platform considered here, with established molecular-beam epitaxy and array-level demonstrations.^4^ The best-supported hybrid examples include MoS_2_/T2SL barrier devices, which reduced dark current by approximately 100-fold at 77 K, and graphene/InAs–GaInSb devices, which supplied photogating and a matched 217-fold LWIR detectivity increase within one experimental study.^6,8^ These results establish useful functions for the 2D layer, but they do not yet show that hybrids outperform the best standalone T2SL focal-plane arrays under common operating conditions.

Transition metal dichalcogenide heterostructures offer complementary capabilities for shorter wavelength detection, with bandgaps tunable through layer number, composition, and interlayer coupling strength.^10^ The work by Zhao *et al.*^10^ demonstrated that MoS_2_/WSe_2_ superlattices exhibit continuously tunable direct bandgaps ranging from 0.14 eV to 0.5 eV depending on the thickness ratio of constituent layers, maintaining type-II alignment across this range. This tunability enables targeting specific spectral bands without requiring entirely different material systems, offering advantages for multispectral detection applications.

Mixed-dimensional heterostructures combining 2D materials with zero-dimensional quantum dots or three-dimensional bulk semiconductors have attracted increasing attention for their ability to leverage the complementary properties of constituent components.^14^ The MoS_2_/PbS quantum dot phototransistors reported by Pak *et al.*^14^ exemplify this approach, utilizing the high mobility of the MoS_2_ channel in conjunction with the strong infrared absorption of lead sulfide quantum dots. The consecutive type-II junctions formed in this configuration enable efficient charge separation while the photogating mechanism provides substantial gain enhancement.

### 2.2 Interface chemistry and bonding characteristics

The electronic properties of heterostructure photodetectors depend critically on the chemical nature and structural quality of interfaces between constituent layers. van der Waals bonding characterizes interfaces in 2D material heterostructures, enabling the formation of atomically sharp junctions without the lattice matching constraints that dominate conventional epitaxial growth.^22^ This weak interlayer coupling preserves the intrinsic electronic properties of individual layers while permitting charge transfer processes governed by band alignment rather than interface states.

The Tang *et al.*^22^ theoretical study of C_3_N/C_3_B bilayer heterostructures explicitly highlighted that despite the weak van der Waals interactions coupling the electron-rich C3N and electron-deficient C3B monolayers, the system supports extremely bright interlayer excitons with binding energies of 0.2–0.4 eV. This finding challenges the conventional assumption that weak interlayer coupling necessarily limits optical activity, demonstrating instead that appropriate band alignment can produce strong interlayer transitions even in van der Waals systems.

Interface evidence must be separated from device inference. The MoS_2_/T2SL barrier reported by Xiao *et al.* produced about a 100-fold reduction in dark current at 77 K and a detectivity of 4 × 10^10^ jones, providing a direct matched-control demonstration.^6^ By contrast, barrier heights inferred from Kelvin-probe measurements, photoluminescence quenching, or idealized calculations establish energetics but do not by themselves prove reduced detector noise. Table 2 therefore states the evidence type and avoids treating qualitative interface sharpness as a quantitative device metric.

**Table 2:** Interface characteristics and band alignment parameters

Interface	Bonding/chemistry	Direct evidence	Device implication	Required validation
MoS_2_/T2SL^6^	vdW transfer; possible residue and vacancy states	Matched 77 K current comparison	∼100× dark-current reduction	Wafer uniformity, noise spectrum, stability
Graphene/T2SL^8^	vdW contact and electrostatic photogating	Matched control under one protocol	217× LWIR D* increase	Separate gain, contact, and absorber contributions
InAs/GaSb T2SL^23,24^	Growth-sequence-dependent InSb-like/mixed bonds	Structural and spectroscopic interface analysis	Offsets and generation centres can shift	Correlate chemistry with leakage and lifetime
T2SL mesa^25,26^	Sulfur or ALD Al_2_O_3_ passivation	Surface-current reduction at 77 K	Lower perimeter leakage	Check hysteresis, fixed charge, ageing
MoS_2_/PbS QDs^14^	TBAI/EDT ligand exchange	Built-in-potential and transient response	Charge separation and 950 µs response	Ligand coverage and ambient stability
InSe/PdSe_2_ (ref. 11)	vdW interface	DFT plus PL/KPFM	Gain-assisted NIR response	Measured trap density and calibrated noise

**Fig. 2:**
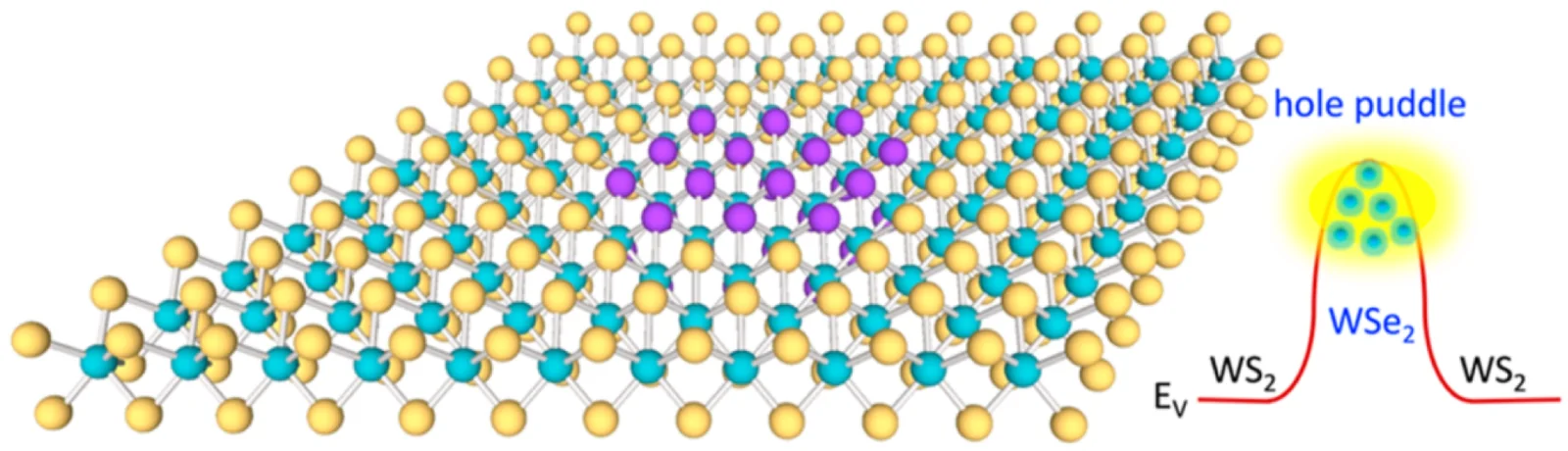
Schematic of WSe_2_ hole puddle formed in WSe_2_ nanodot embedded in WS_2_ matrix due to the type-II band alignment at the WSe_2_/WS_2_ heterojunction.

The formation of charge puddles at type-II heterointerfaces represents a distinct interface phenomenon with significant implications for photodetector performance.^12^ Tsai *et al.*^12^ demonstrated that WSe_2_ nanodots embedded in WS_2_ form localized hole puddles upon photoexcitation, with the built-in field at the heterojunction separating electrons and holes (Fig. 2). These charge puddles provide an effective photogating mechanism that enhances responsivity while the ultrafast intralayer excitonic dynamics in the WSe_2_/WS_2_ system reduce response time by five orders of magnitude compared to trap-limited devices.

**Fig. 3:**
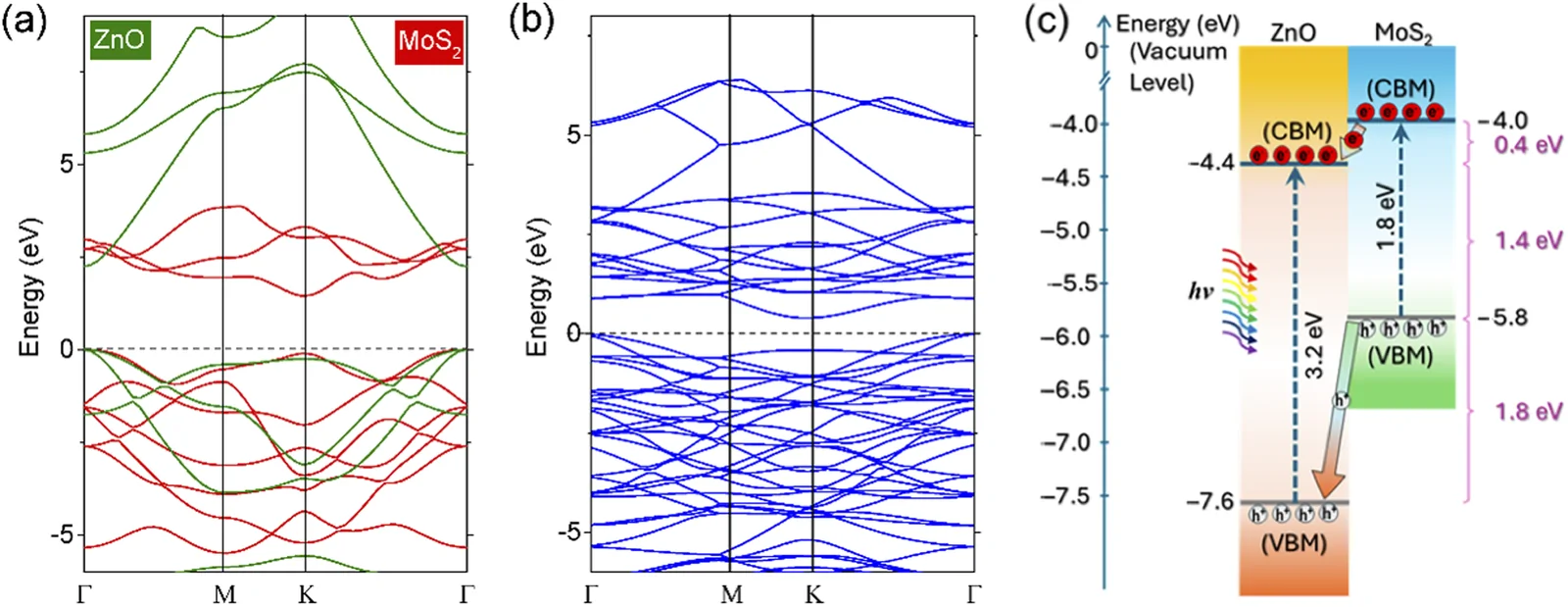
Simulated energy bandgaps of (a) ZnO and MoS_2_ monolayers and (b) ZnO–MoS_2_ heterostructure. The valence band maximum (VBM) is set to zero. (c) Schematic presentation of type II heterostructure formed of ZnO and MoS_2_. CBM: conduction band minimum. Adapted/reproduced from ref. 27 with permission from MDPI, copyright 2025.

Chemical composition at an interface can alter both band offsets and noise. Transfer residues, oxidation, sulfur vacancies, interdiffusion, and growth-sequence-dependent InSb-like or mixed bonds can introduce fixed charge or generation–recombination centers even when microscopy shows a continuous junction.^23,24^ Accordingly, spectroscopy or microscopy should be paired with electrical observables such as surface leakage, capacitance–voltage hysteresis, low-frequency noise, and bias-dependent dark current. Candidate treatments identified in monolayer transistors or photoluminescence studies remain hypotheses for T2SL/2D detectors until the same device is measured before and after treatment (Fig. 3).

### 2.3 Band engineering strategies

First-principles calculations are valuable for screening band alignment, charge redistribution, defects, strain, and candidate passivation reactions, but their output must be matched to the experimental observable. Semilocal Kohn–Sham gaps are not quasiparticle gaps; hybrid functionals or GW calculations are generally required for quantitative band edges, while optical onsets in weakly screened 2D systems may additionally require a Bethe–Salpeter treatment of excitons.^28,29^ Absolute offsets also depend on slab thickness, vacuum spacing, *k*-point sampling, dipole corrections, Coulomb truncation, spin–orbit coupling, and the selected atomic registry. Consequently, a predicted type-II alignment is best interpreted as a mechanistic hypothesis unless these convergence choices and uncertainties are reported.

Yu *et al.*^30^ extended band engineering concepts to SnX_2_/ZnS heterostructures, demonstrating through DFT calculations that the bandgap can be continuously tuned from 0 to 0.97 eV by varying the composition of SnS_2(1−*η*)_Se_2*η*_ alloy layers while maintaining type-II alignment characteristics. This composition-dependent tunability enables photodetection across wavelengths from 1.2 µm to 10 µm, covering substantial portions of the SWIR, MWIR, and LWIR bands within a single material platform.^30^ The wide tuning range represents a significant advantage over systems where spectral coverage requires entirely different material combinations.

Defect engineering can tune calculated bands, but the same defect may also become a recombination or noise center. For example, vacancy-induced sub-gap states can extend absorption while increasing trap-assisted generation and 1/*f* noise.^31^ The relevant experimental test is therefore not spectral extension alone but a joint measurement of external quantum efficiency, calibrated noise, response time, stability, and defect density at a defined chemical potential. Deliberate vacancy creation should be distinguished from uncontrolled process damage.

**Fig. 4:**
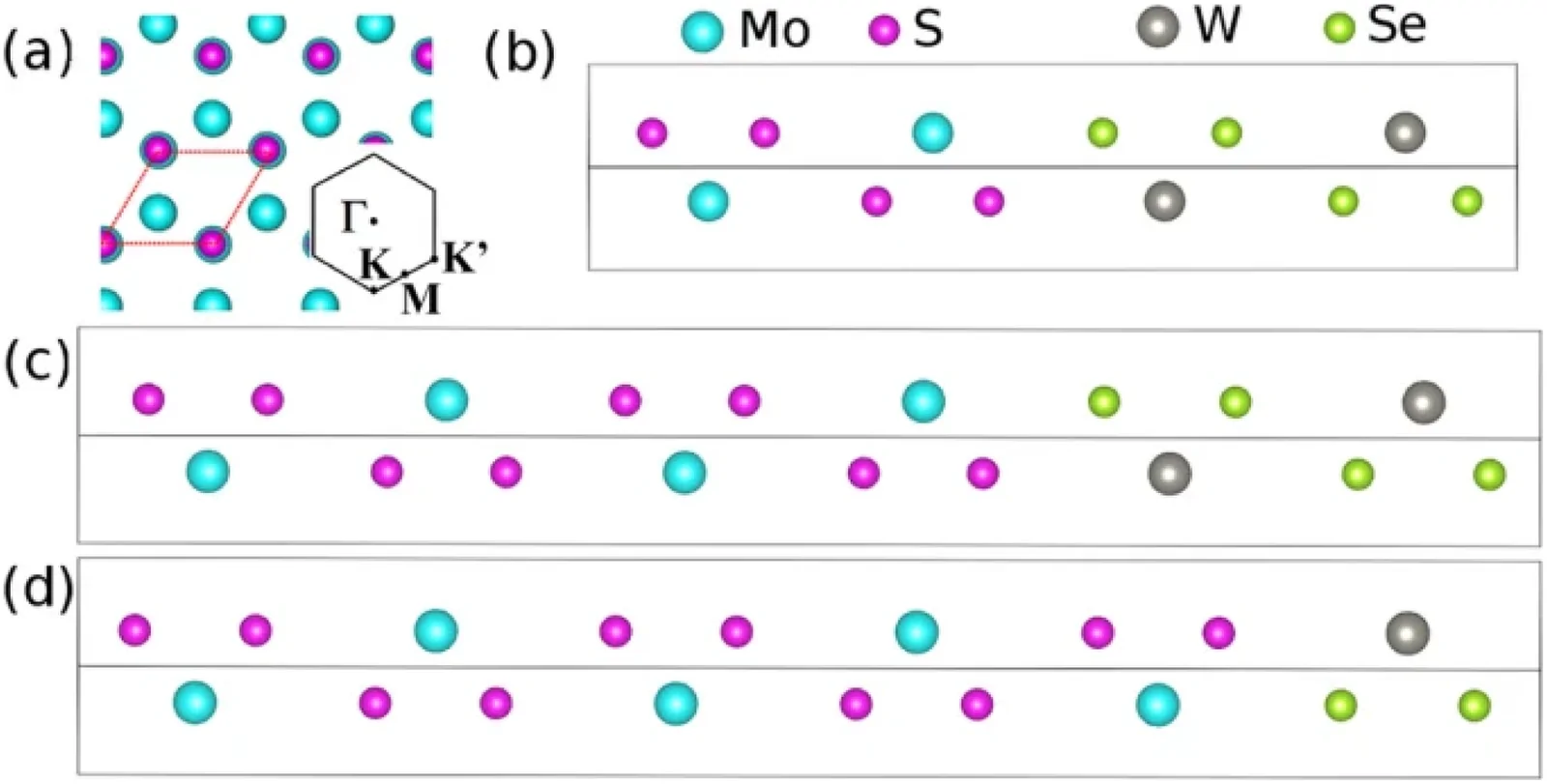
(a) Top view of a superlattice as indicated by solid lines and its associated first Brillouin zone; side views of (b) the 1 : 1 superlattice where MoS_2_ : WSe_2_ equals to 1 : 1; (c) the 2 : 1 superlattice where MoS_2_ : WSe_2_ equals to 2 : 1; (d) the 5 : 1 monolayer superlattice where all MoS_2_ and WSe_2_ bilayers were replaced by the corresponding monolayers. Adapted/reproduced from ref. 10 with permission from Springer Nature, copyright 2015.

Strain effects represent a critical consideration for practical heterostructure devices, particularly when combining materials with different lattice parameters or thermal expansion coefficients.^32^ Zhao *et al.*^33^ found that in-plane strain induced by lattice mismatch in TMDC superlattices can drive transitions between indirect and direct bandgap character, as well as semiconductor-to-metal transitions at sufficiently large strains (Fig. 4). The band offset modifications accompanying strain indicate that careful attention to mechanical constraints is necessary to preserve desired electronic properties in fabricated devices.^34,35^

The manipulation of interlayer exciton (IX) properties through applied electric fields provides dynamic control over effective bandgaps in operating devices.^36,37^ Zhang *et al.*^38^ demonstrated that IX emission in WSe_2_/InSe heterostructures can be electrically tuned by 180 meV through the Stark effect, enabling voltage-controlled spectral response. The IX binding energy of 43 ± 5 meV, extracted from temperature-dependent photoluminescence, indicates stable exciton populations at room temperature suitable for practical photodetection applications.^39,40^

## 3. Carrier transport mechanisms and dynamics

### 3.1 Interlayer charge transfer processes

The efficiency of type-II heterostructure photodetectors fundamentally depends on the mechanisms and rates of charge transfer across layer interfaces following photoexcitation.^41^ In type-II systems, photogenerated electron–hole pairs undergo spatial separation driven by band offsets, with electrons transferring to one material and holes to the adjacent layer.^42^ This separation suppresses direct recombination and extends carrier lifetimes, but must occur sufficiently rapidly to compete with alternative relaxation pathways.

Asaithambi *et al.* used steady-state and time-resolved spectroscopy to resolve charge transfer in InAs@ZnSe core–shell quantum-dot/MoS_2_ heterostructures.^13^ Under excitation of both components, holes transferred toward the InAs@ZnSe nanostructures, whereas selective quantum-dot excitation revealed electron transfer toward MoS_2_. The device showed broadband response from 300 to 850 nm. This system is a 0D/2D sensitized junction rather than a T2SL/2D hybrid, and its high gain should be interpreted together with the measured transient and noise conditions reported in the primary study.

The formation of interlayer excitons in type-II heterostructures represents an intermediate state in the charge transfer process with distinct signatures in optical spectroscopy.^37,43,44^ Zhang *et al.*^38^ constructed WSe_2_/InSe van der Waals heterostructures hosting momentum-direct interlayer excitons with near-infrared emission at room temperature. First-principles calculations verified the band alignment supporting direct interlayer transitions, and the observed IX binding energy of 43 meV confirms the spatially indirect nature of these quasiparticles.^45–47^

Wu *et al.* demonstrated a vertical graphene/black-phosphorus/MoS_2_/graphene photodetector in which the short transport distance supported both broadband response and rapid collection.^48^ The wavelength-specific results must be kept separate: the peak specific detectivity was 2.38 × 10^11^ jones at 3600 nm, whereas the rise and decay times were 10.4 and 15.6 ns, respectively, under 1550 nm excitation. This architecture is a 2D van der Waals junction rather than an epitaxial T2SL/2D hybrid. Its speed illustrates the advantage of a vertical channel; it should not be combined with the 3600 nm detectivity into a single operating point (Table 3).

**Table 3:** Carrier transport parameters in type-II heterostructures

System	Mechanism	Quantitative result	Conditions	Comparison limit
MoS_2_/T2SL^6^	Carrier-selective barrier	∼100× lower dark current; D* = 4 × 10^10^ jones	77 K; matched control	Device-level, not array yield
Graphene/T2SL^8^	Photogating	D* 1.69 × 10^6^ → 3.66 × 10^8^ jones	77 K; 8–12 µm; matched	Gain may affect bandwidth/linearity
Gr/BP/MoS_2_/Gr^48^	Vertical collection	D* = 2.38 × 10^11^ jones at 3600 nm; 10.4/15.6 ns at 1550 nm	Room temperature	D* and speed are at different wavelengths
InSe/PdSe_2_ (ref. 11)	Gain-assisted interlayer transport	*R* = 58.8 A W^−1^; EQE = 4660% at 1650 nm	Reported device conditions	EQE implies ∼46.6e^−^ per photon
MoS_2_/PbS QDs^14^	Photogating/built-in potential	950 µs	850 nm transient	Not a T2SL hybrid
WSe_2_/WS_2_ (ref. 12)	Charge-puddle photogating	Gain with accelerated transient	Visible excitation	Mechanism transferable; spectrum differs
M-barrier T2SL^49^	Barrier + Fabry–Perot cavity	D* = 5 × 10^11^ at 7.9 µm	77 K; BLIP test pixel	Optimized standalone benchmark

### 3.2 Photogating effects and gain mechanisms

Photogating represents a powerful gain mechanism in 2D heterostructure photodetectors wherein trapped or accumulated charges modulate the channel conductance, producing amplified photocurrent response.^50–52^ This effect typically arises from the spatial separation of photogenerated carriers at type-II interfaces, where one carrier type becomes localized while the opposite carrier recirculates through the channel during the trapped carrier lifetime.^53^

Fukushima *et al.* coupled graphene to an InAs–GaInSb T2SL so that photocarriers in the superlattice gated graphene transport.^8^ At 77 K and with an 8–12 µm bandpass, the optimized hybrid yielded D* = 3.66 × 10^8^ jones compared with 1.69 × 10^6^ jones for the matched T2SL control, a 217-fold enhancement. This within-study comparison is strong evidence for hybrid photogating, but the absolute D* is not a field-wide record and should not be compared directly with values obtained at other temperatures, wavelengths, areas, or noise bandwidths.

Tsai *et al.* formed WSe_2_ nanodots within WS_2_ so that photogenerated holes accumulated in localized puddles and gated the surrounding channel.^12^ The authors reported photoconductive gain of about 700 electrons per photon and responsivity of 300 A W^−1^, while the zero-gate transient was much faster than in their selected trap-limited reference device. The mechanism differs from persistent deep-trap photogating because rapid intralayer excitonic dynamics and the engineered lateral junction govern carrier release; nevertheless, its visible-excitation conditions do not establish infrared T2SL performance.

The graphene heterojunction architecture reported by Tsai *et al.* utilizing alternating WS_2_–WSe_2_ strip superstructures demonstrates another approach to overcoming the responsivity-speed trade-off.^54^ Upon illumination, the n^+^-WS_2_ and p^+^-WSe_2_ strips create alternating electron and hole conduction channels in the graphene, enabling responsivity of 1.7 × 10^7^ mA W^−1^ with response time of 3–4 µs.^54^ The superstructure design extends photodetection from near-ultraviolet through mid-infrared wavelengths while maintaining fast temporal response.

### 3.3 Dark current suppression strategies

Minimizing dark current represents a critical challenge for infrared photodetectors, particularly at longer wavelengths where thermally generated carriers in narrow-bandgap materials produce substantial noise.^55,56^ Type-II heterostructures offer intrinsic advantages for dark current suppression through spatial separation of carriers and engineered barriers that impede thermal generation without blocking photogenerated carriers.^57,58^

Xiao *et al.* integrated MoS_2_ with a T2SL as a wide-bandgap van der Waals barrier.^6^ At 77 K, the hybrid showed approximately two orders of magnitude lower dark current than its barrier-free control and reached D* = 4 × 10^10^ jones. The result supports carrier-selective filtering by the 2D layer; however, it remains a device-level demonstration rather than evidence of wafer-scale transfer uniformity or focal-plane-array yield.

A separate conference report investigated low-noise T2SL detector design, but the available record does not provide a sufficiently transparent matched device/control dataset to support the previously quoted 1000-fold reduction, 6 × 10^−6^ A cm^−2^, and 5 × 10^9^ jones as one verified operating point.^59^ Those numbers are therefore omitted from the cross-platform comparison. The robust conclusion is narrower: barrier height, interface trap density, and contact geometry must be optimized together, and the reduction factor should be reported with temperature, bias, area, and the exact control structure.

The graphene diode configuration reported by Fukushima *et al.* offers an alternative approach wherein the rectifying characteristics of the graphene/T2SL junction inherently suppress dark current.^60^ The dark current in graphene diode devices was reduced to less than 1% compared to graphene field-effect transistor configurations, while the photogating effect provided significant responsivity enhancement. This combination of low noise and high gain represents an advantageous operating regime for sensitive photodetection.

Huang *et al.* reported 77 K background-limited dual-band T2SL test pixels with D* values of 5 × 10^11^ jones at 7.9 µm and 1 × 10^11^ jones at 10.2 µm.^49^ These values arose from the combined M-barrier and Fabry–Perot cavity design, not from the electronic barrier alone. They illustrate the performance of an optimized standalone T2SL control class and provide essential context for claims of hybrid superiority (Table 4).

**Table 4:** Dark current reduction strategies and performance outcomes

Strategy	System	Verified outcome	Conditions/control	Qualification
vdW barrier	MoS_2_/T2SL^6^	∼100× lower dark current; D* = 4 × 10^10^	77 K; matched barrier-free device	Transfer uniformity not established
Photogating	Graphene/T2SL^8^	217× D* enhancement	77 K; 8–12 µm; matched	Within-study result, not field record
Vertical transport	Gr/BP/MoS_2_/Gr^48^	10.4/15.6 ns rise/fall	1550 nm; room temperature	Separate from 3600 nm D*
M-barrier + cavity	Standalone T2SL^49^	5 × 10^11^ at 7.9 µm; 1 × 10^11^ at 10.2 µm	77 K; BLIP test pixels	Electronic and optical effects coupled
Surface passivation	T2SL mesa^25,26^	Reduced perimeter leakage	77 K; process-specific	Fixed charge and ageing must be tested

### 3.4 Ultrafast carrier dynamics

The temporal response of photodetectors is governed by carrier generation, transport, and collection processes, with each step potentially limiting the overall speed.^48^ Hot carrier dynamics in plasmonic heterostructures and ballistic transport in 2D materials offer pathways toward ultrafast photodetection that circumvent conventional limitations.

Yu *et al.*^61^ reviewed hot-carrier engineering strategies for 2D integrated infrared optoelectronics, emphasizing that hot carriers exhibit ultrafast interfacial transfer and ballistic transport characteristics distinguishing them from conventional band-edge carriers. The harvesting of hot carriers prior to thermalization minimizes energy dissipation while enabling detection of sub-bandgap photons beyond the spectral limitations of the semiconductor absorber. These mechanisms provide unprecedented opportunities for high-speed photoelectric conversion in infrared devices.

**Fig. 5:**
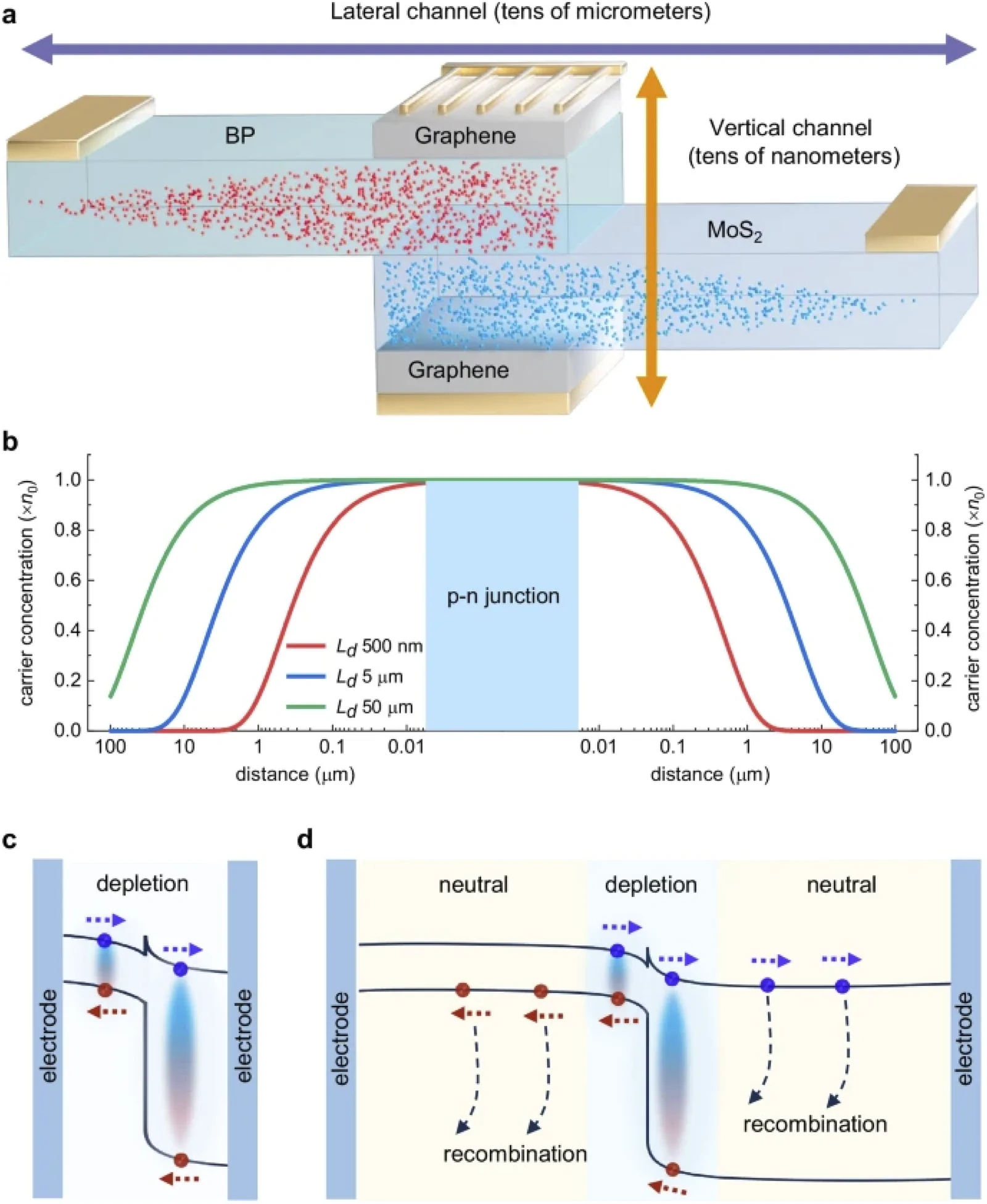
(a) Schematic diagram illustrating the diffusion process of photogenerated charge carriers in both vertical and lateral channel devices, with incident light directed from above onto the overlapped heterojunction region. The red and blue spheres represent holes and electrons, respectively. (b) Steady-state photogenerated charge carrier distribution as a function of distance from the p–n junction when excess electrons and holes are exclusively generated at the p–n junction region. *L*_d_ represents the diffusion length of the charge carriers. *n*_0_ refers to the photogenerated carrier concentration at the p–n junction region. (c and d) Schematic energy band diagram showing the electron–hole pair generation, separation, diffusion, and recombination processes in both vertical (c) and lateral channel devices (d). The red and blue dashed arrows indicate the transport directions of photogenerated holes and electrons, respectively. The blue and orange shaded areas correspond to the depletion and neutral regions, respectively. Adapted/reproduced from ref. 48 with permission from Springer Nature, copyright 2025.

The vertical-channel device of Wu *et al.* achieved rise and decay times of 10.4 and 15.6 ns, respectively, under 1550 nm excitation.^48^ The separate detectivity maximum occurred at 3600 nm. The nanosecond transient result is therefore cited as a speed benchmark at 1550 nm, not as the response time corresponding to the peak mid-infrared D* (Fig. 5).

## 4. Performance enhancement strategies

### 4.1 Surface plasmon resonance enhancement

Optical enhancement can raise absorbed photon flux without invoking electronic gain, but improvements should be demonstrated against a geometrically and electrically matched control. Plasmonic structures can concentrate fields in an ultrathin absorber; their benefits must be weighed against metal loss, process variation, spectral narrowing, and possible heating.^62^

In the WS_2_/MoS_2_ study, the infrared response at 1030 nm was attributed to interlayer coupling and plasmon-assisted absorption, whereas the isolated constituents showed no comparable response.^62^ The mechanism is informative for 2D/2D junctions, but it is not evidence for a T2SL/2D hybrid. A quantitative claim requires the raw responsivity and noise of matched devices with and without the optical structure, measured at the same bias and modulation frequency.

**Fig. 6:**
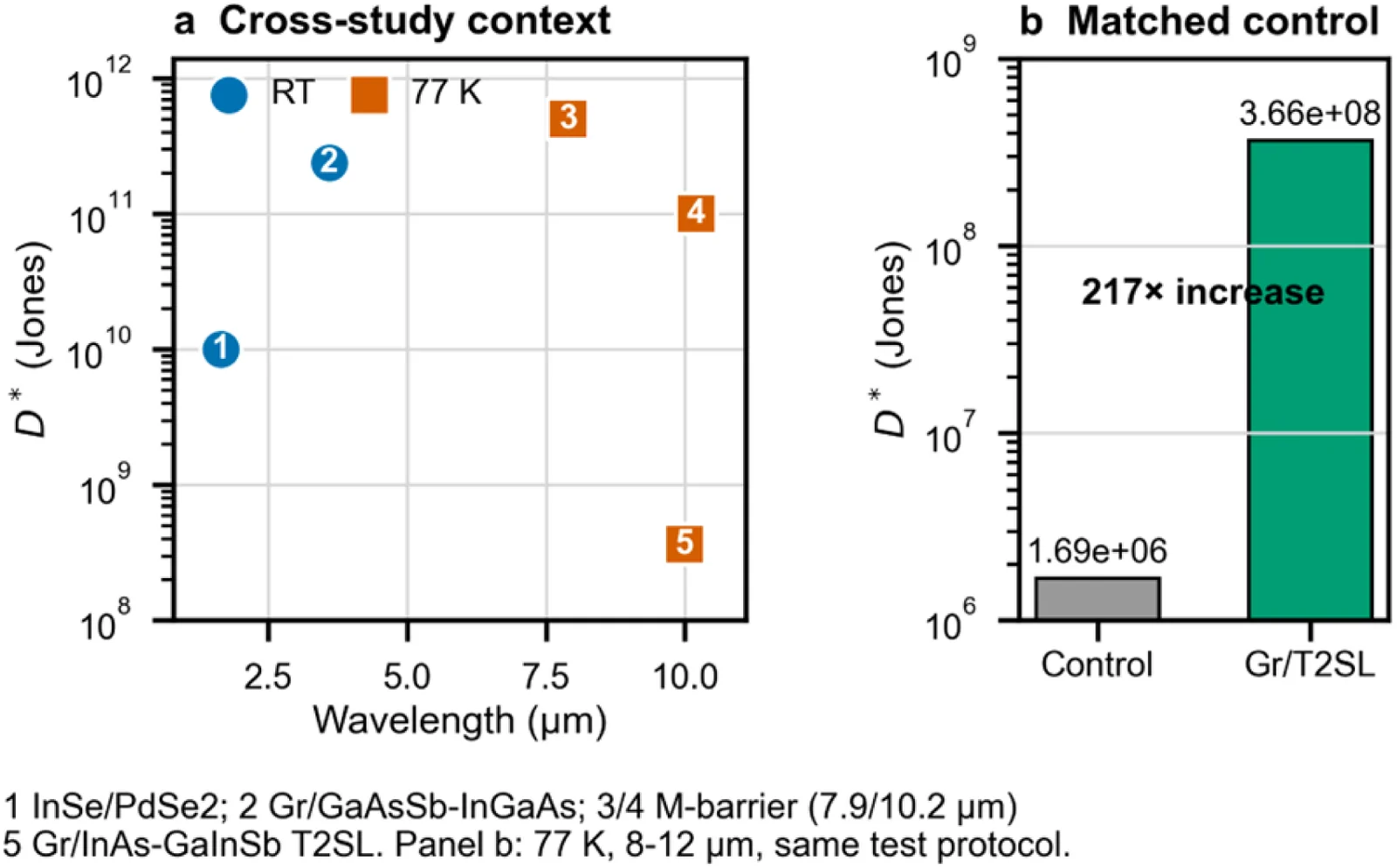
Condition-aware comparison of selected infrared-detector results. (a) Representative D* values are labelled by wavelength and temperature; cross-study points provide context rather than a rank order because device area, bandwidth, bias, and noise extraction differ. (b) Matched 77 K, 8–12 µm comparison from Fukushima *et al.* shows D* = 1.69 × 10^6^ jones for the T2SL control and 3.66 × 10^8^ jones for graphene/T2SL, corresponding to a 217-fold enhancement. Adapted/reproduced from ref. 8 with permission from Optica Publishing Group, copyright 2024.

Fig. 6 replaces an earlier conceptual image that did not correspond to its caption. Panel a plots only primary-report D* values for which wavelength and temperature were identified, and the symbols deliberately separate room-temperature and 77 K data. Panel b isolates the graphene/T2SL result for which the hybrid and T2SL control were tested in the same study. This presentation prevents disparate temperatures, spectral bands, and noise-extraction methods from being mistaken for a performance ranking.^8,9,11,48,49^

### 4.2 Metasurface and optical engineering

Beyond localized plasmons, periodic metasurface structures offer additional degrees of freedom for manipulating light coupling into heterostructure absorbers.^63^ Mie dielectric metasurfaces provide resonant enhancement without the ohmic losses associated with metallic plasmonic structures, potentially offering superior performance for infrared applications.

Xiao *et al.*^63^ developed pixel-integrated long-wavelength multispectral T2SL detectors based on Mie dielectric metasurfaces incorporating graphene-assisted depletion tuning. The graphene-assisted depletion Mie metasurface structure (GAMS) enables 340 nm electrical dynamic tuning of the spectral response while achieving detectivity of 5 × 10^10^ jones. This combination of high performance and spectral agility addresses requirements for dynamic multispectral imaging in applications ranging from space exploration to environmental monitoring.

The Fabry–Perot resonance incorporated in the M-barrier T2SL imagers reported by Huang *et al.*^49^ demonstrates that optical cavity effects can compensate for thin absorber regions. Despite using only 2 µm absorbing layers, the devices achieve quantum efficiencies sufficient for background-limited operation, with noise equivalent temperature differences of approximately 20 milli-Kelvin for both spectral channels. This approach enables the use of high-quality thin epitaxial layers while maintaining competitive absorption efficiency.

### 4.3 Interlayer exciton engineering

Interlayer excitons formed at type-II heterointerfaces possess characteristics distinct from intralayer excitons that can be exploited for infrared detection.^22^ The spatial separation of electron and hole wavefunctions reduces oscillator strength but extends radiative lifetime and enables absorption at energies below the constituent material bandgaps.

Tang *et al.* predicted bright interlayer excitons with binding energies of 0.2–0.4 eV in C_3_N/C_3_B bilayers using many-body perturbation theory.^22^ The result shows that spatially separated electron and hole states need not have negligible oscillator strength. However, experimental synthesis, disorder, dielectric environment, non-radiative loss, and calibrated detector noise remain unverified for this specific proposal, so it is presented as a candidate mid- to far-infrared absorber rather than a demonstrated room-temperature detector.

Lukman *et al.*^64^ demonstrated infrared photodetection enabled by interlayer excitons in WS_2_/HfS_2_ heterostructures, with the absorption band tunable and extendable to 20 µm under modest electric fields. *Ab initio* simulations revealed that sizeable charge delocalization and accumulation at the interface result in greatly enhanced oscillator strength compared to expectations for spatially indirect transitions.^65^ This enhancement enables high responsivity despite the indirect nature of the interlayer absorption process.

### 4.4 Ferroelectric and pyroelectric enhancement

Ferroelectric materials integrated with 2D heterostructures introduce additional functionality through spontaneous polarization that can enhance carrier separation and collection. The ferroelectric polarization creates internal electric fields that supplement the built-in fields at type-II interfaces, potentially improving both responsivity and response speed.^66^

Guo *et al.*^67^ identified ferroelectric-enhanced designs as one of four principal strategies for improving 2D infrared photodetector performance, alongside type-II band alignments, photogating enhancement, and surface plasmon designs. The ferroelectric polarization provides persistent electric fields that facilitate carrier separation without requiring external bias, enabling self-powered operation modes attractive for remote sensing and portable applications.^68^

The ZnO–MoS_2_ junction reported by Zhou *et al.* displayed light-dependent current–voltage hysteresis and self-powered response.^27^ Because interfacial trapping and ionic or defect redistribution can also generate hysteresis, that observation is described here as ferroelectric-like rather than proof of switchable ferroelectric polarization. Polarization–electric-field measurements and retention/endurance controls would be required before assigning a ferroelectric enhancement mechanism.

### 4.5 Chemical interface engineering and passivation

Chemical engineering complements geometric and electrostatic design by changing trap density, fixed charge, band bending, and environmental stability. InAs/GaSb T2SL mesas treated with ALD Al_2_O_3_ showed approximately an order-of-magnitude dark-current reduction at 77 K, while electrochemical sulfur treatment increased surface resistivity and improved detectivity in separate T2SL studies.^25,26^ These results support passivation as a route to suppress perimeter generation–recombination, but neither treatment can be transferred uncritically to a 2D/T2SL stack because ALD nucleation, fixed charge, and sulfur-vacancy chemistry may alter the 2D layer.

At epitaxial interfaces, growth sequence and shutter timing determine InSb-like, GaAs-like, or intermixed bonds and thereby modify band offsets and recombination centres.^23,24^ At transferred van der Waals interfaces, solvent residue, adsorbed water, oxidation, wrinkles, and chalcogen vacancies can dominate the nominally dangling-bond-free junction. Ligand exchange in MoS_2_/PbS quantum-dot devices illustrates chemical control of built-in potential and carrier residence time.^14^ For T2SL/2D hybrids, promising treatments include low-damage cleaning, encapsulation, seeded ALD, molecular doping, and vacancy passivation, but each requires a matched untreated control.

A credible chemical-modification study should report surface composition and bonding (XPS or related spectroscopy), morphology, band alignment, trap or hysteresis metrics, full noise spectra, response speed, and accelerated ageing before and after treatment. Improvements shown only in monolayer photoluminescence or field-effect mobility identify candidates; they do not establish infrared-detector performance until validated in the complete heterostructure.^69^

### 4.6 Comparative analysis of enhancement approaches

Table 5 compares enhancement mechanisms by the metric they are expected to change and by their characteristic penalty. Photogating can raise responsivity and EQE above 100% because one absorbed photon modulates the channel long enough for multiple carriers to circulate; the resulting EQE is a gain metric, not a photon-conversion probability. Barrier engineering primarily targets dark-current and noise reduction, vertical transport targets transit time, and optical resonators target absorption. No strategy can be called superior without a matched control and a common set of wavelength, temperature, bias, bandwidth, and speed measurements.

**Table 5:** Summary of performance enhancement strategies

Enhancement strategy	Primary target	Representative evidence	Characteristic trade-off	Minimum comparison set
vdW barrier	Dark current/noise	MoS_2_/T2SL: ∼100× lower dark current^6^	Series resistance, transfer defects	Matched absorber, area, bias, *T*
Photogating	Responsivity/EQE	Graphene/T2SL: 217× D*^8^	Bandwidth, linearity, 1/*f* noise	*R*, D*, transient, noise spectrum
Vertical channel	Response time	10.4/15.6 ns at 1550 nm (ref. 48)	Absorption volume/contact effects	Same wavelength and bandwidth
Charge puddles	Gain with faster release	WSe_2_/WS_2_ (ref. 12)	Trap distribution and drift	Gain, fall time, hysteresis
Plasmonic coupling	Absorption	WS_2_/MoS_2_ response at 1030 nm (ref. 62)	Metal loss/heating	Matched optical geometry
Dielectric metasurface	Spectral tuning	GAMS/T2SL: 340 nm tuning^70^	Fabrication tolerance	Angle, polarization, NETD
Chemical passivation	Surface leakage	S and ALD-treated T2SL mesas^25,26^	Fixed charge, long-term drift	Before/after noise and ageing
M-barrier + cavity	Noise plus absorption	Dual-band BLIP pixels^49^	Epitaxial/optical complexity	Separate electronic/optical controls

Surface plasmon enhancement offers broadband responsivity improvement without fundamentally altering the electronic transport characteristics, but introduces additional fabrication complexity and potential losses at longer wavelengths. The dielectric metasurface approach avoids plasmonic losses while enabling spectral selectivity and tunability, representing an attractive compromise for applications requiring specific wavelength targeting.^63^

Barrier engineering for dark current suppression directly improves signal-to-noise ratio without affecting the photoresponse mechanism, making it broadly compatible with other enhancement strategies.^6^ The combination of barrier structures with photogating or plasmonic enhancement could potentially yield multiplicative improvements, though experimental demonstrations of such integrated approaches remain limited.

## 5. Device performance and applications

### 5.1 Spectral coverage and detectivity achievements

Reported performance varies with spectral band, temperature, bias, area, modulation frequency, and noise methodology. Fig. 6 therefore separates contextual cross-study values from a matched-control comparison. Specific detectivity is useful only when the noise current is measured or physically justified; D* values reconstructed from shot noise alone can be strongly inflated when 1/*f*, generation–recombination, contact, or readout noise dominates.^9^

For graphene/InAs–GaInSb T2SL devices tested by Fukushima *et al.* at 77 K with an 8–12 µm bandpass, the hybrid yielded NEP = 4.09 × 10^−12^ W Hz^−1/2^ and D* = 3.66 × 10^8^ jones; the matched T2SL control yielded NEP = 8.87 × 10^−10^ W Hz^−1/2^ and D* = 1.69 × 10^6^ jones.^8^ The corresponding 217-fold enhancement is an internally controlled result. The hybrid value is not described as the highest across the field because higher values elsewhere were obtained under different temperature, wavelength, architecture, and noise conditions.

Wu *et al.* reported a peak D* of 2.38 × 10^11^ jones at 3600 nm and NEP = 1.72 × 10^−14^ W Hz^−1/2^ at the same wavelength for a room-temperature vertical 2D heterostructure.^48^ The ultraviolet-to-mid-infrared spectral span is notable, but it belongs to a 2D/2D architecture and should not be presented as a T2SL/2D result.

Ahmad *et al.* reported an InSe/PdSe_2_ van der Waals photodetector with responsivity of 58.8 A W^−1^, D* = 1 × 10^10^ jones, and a gain-assisted EQE of 4660% at 1650 nm.^11^ An EQE of 4660% corresponds to approximately 46.6 collected electrons per incident photon and therefore signals photoconductive or photogating gain; it must not be interpreted as a passive conversion efficiency exceeding unity.

### 5.2 Response time performance

Temporal response characteristics determine the suitability of photodetectors for applications requiring fast signal acquisition, including high-speed imaging and optical communications. The trade-off between responsivity and response speed represents a central challenge in photodetector design, particularly for devices employing gain mechanisms that rely on carrier trapping or accumulation.^54^

The fastest transient cited here is the 10.4 ns rise and 15.6 ns decay measured at 1550 nm in the vertical graphene/BP/MoS_2_/graphene device.^48^ Gain-assisted devices are compared separately because a long trap lifetime can raise responsivity while reducing bandwidth. A complete speed comparison should report the optical pulse width, electrical bandwidth, bias, active area, and whether the stated value is rise time, fall time, or a fitted lifetime.

**Fig. 7:**
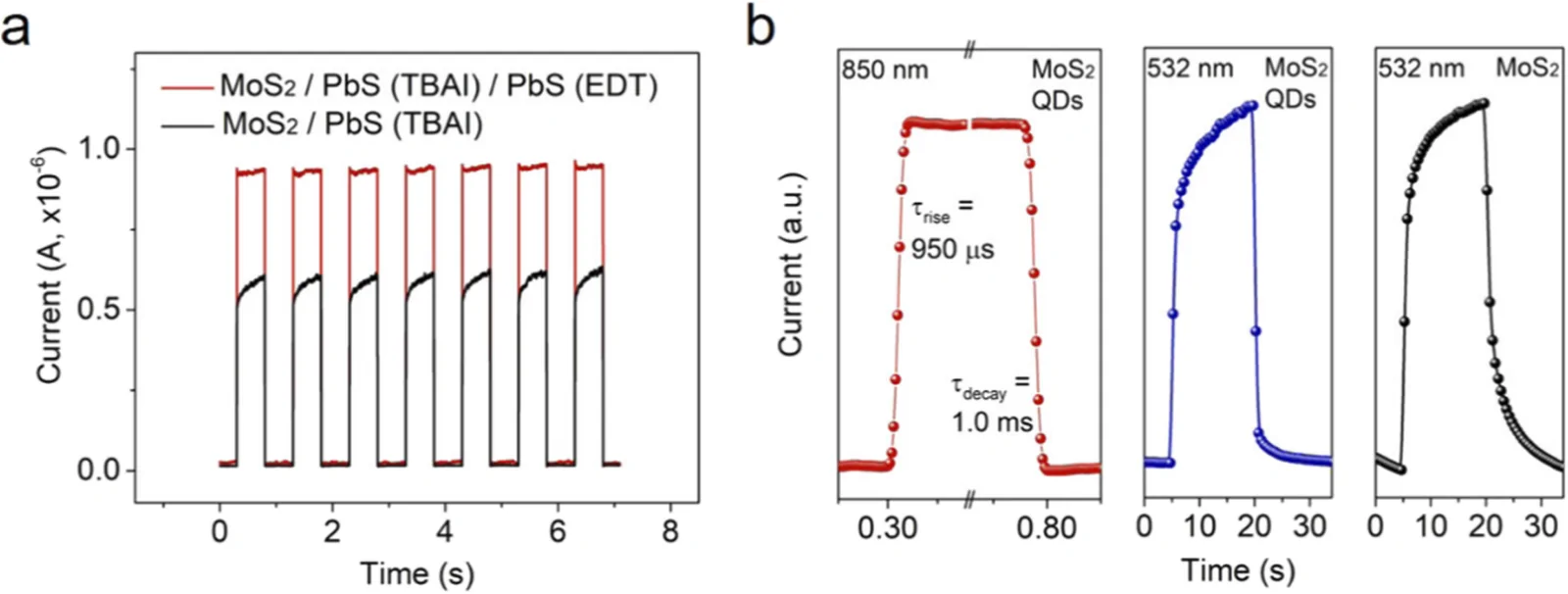
Photoresponse of the MoS_2_/PbS QDs devices. (a) Temporal response of the photocurrent in the MoS_2_/TBAI and MoS_2_/TBAI/EDT devices under 850 nm laser illumination with *V*_d_ = 1 V, *V*_g_ = 0 V, *P*_laser_ = 200 nW. (b) Temporal response of the photocurrent showing a distinctly different response time when the incident energy (850 or 532 nm) is either smaller or larger than the band gap of the MoS_2_ monolayer.^14^ Adapted/reproduced from ref. 14 with permission from ACS, copyright 2018.

The MoS_2_/PbS QD phototransistors reported by Pak *et al.*^14^ achieved response times of 950 µs, faster by orders of magnitude than previously reported 2D/QD phototransistors. The improvement arises from the modulation of built-in potential across the consecutive type-II junctions, which enhances charge separation efficiency and reduces carrier accumulation (Fig. 7). The detectivity of 1 × 10^11^ jones achieved alongside this fast response demonstrates that the trade-off between gain and speed can be favorably managed through junction engineering.

For polarization-sensitive detection, the 2H-MoTe_2/1_T′-MoTe_2_/MoSe_2_ heterostructure devices reported by Pan *et al.*^71^ demonstrated response times of 13 ms rise and 10 ms fall with photocurrent anisotropy ratio of 55. The self-powered operation without external bias or gate voltage, combined with responsivity of 0.76 A W^−1^ and external quantum efficiency of 71%, makes this architecture attractive for polarimetric imaging applications.

### 5.3 Room temperature operation

Room-temperature operation reduces cooling burden but does not by itself establish system readiness. Colloidal-quantum-dot detectors now provide important alternatives, including PbS/HgTe imaging extending toward 5 µm and Ag_2_Te short-wave-infrared detectors with room-temperature D* near 10^12^ jones and bandwidth above 0.1 MHz.^17,18^ QWIPs/QDIPs offer mature epitaxial uniformity and spectral design but often require cooling because of thermionic dark current, whereas nBn and related unipolar barriers suppress majority-carrier current without a depletion region in the absorber.^16,19–21^ 2D junctions add gate tunability, flexible integration, and low capacitance, but their small-area record values should be compared with array uniformity, NETD, yield, and stability rather than D* alone.

The C_3_N/C_3_B bilayer heterostructures predicted by Tang *et al.*^22^ support interlayer excitons with binding energies of 0.2–0.4 eV, sufficient to maintain stable exciton populations at room temperature for mid- to far-infrared detection. While experimental demonstration of these theoretically predicted properties remains pending, the computational results indicate a viable pathway toward room-temperature terahertz detection using atomically thin materials.

**Fig. 8:**
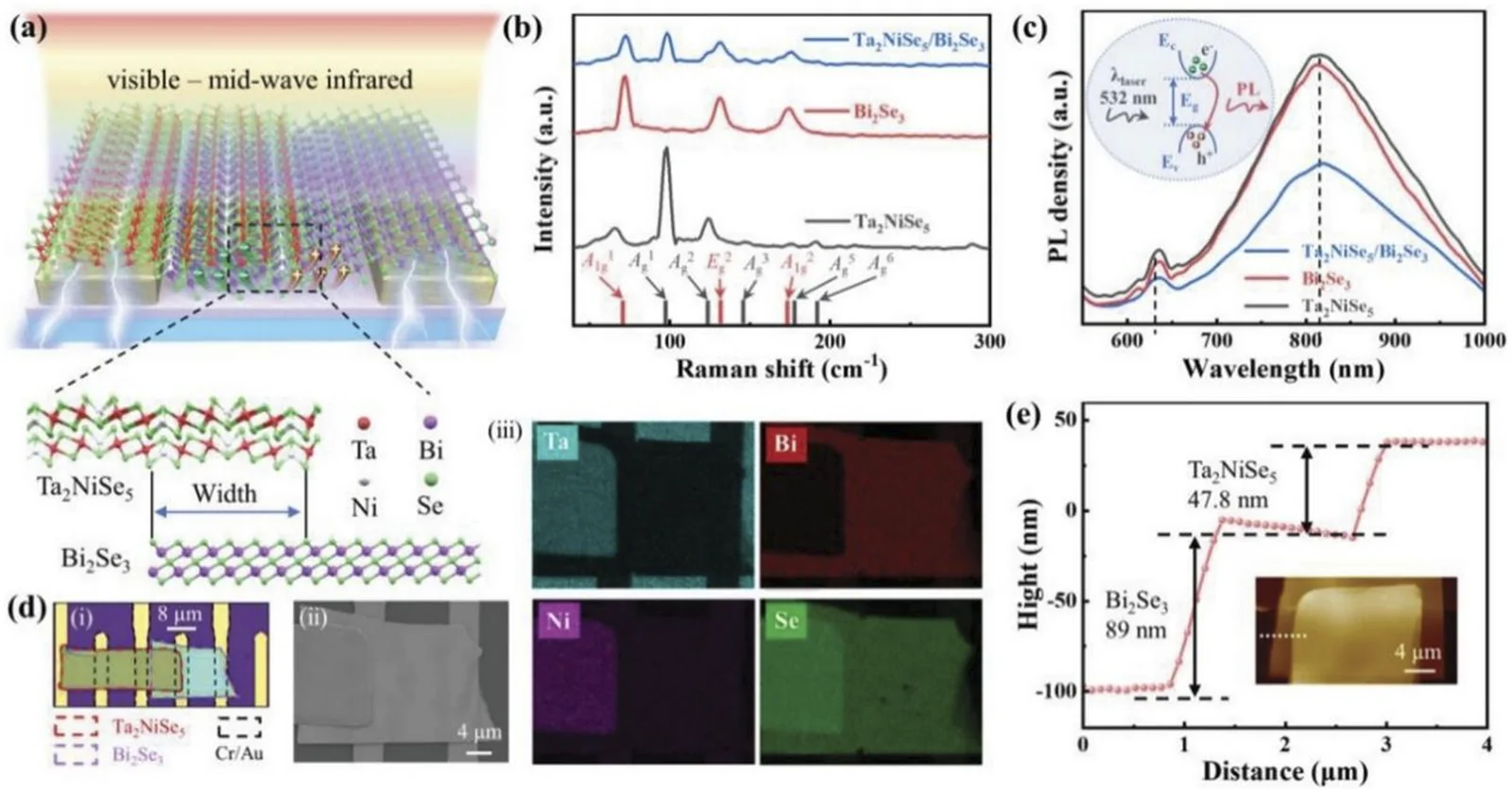
Characterizations of the Ta_2_NiSe_5_/Bi_2_Se_3_ heterostructure on the SiO_2_/Si substrate. (a) A structural diagram of the Ta_2_NiSe_5_/Bi_2_Se_3_ vdWs heterojunction photodetector and atomic structure of Ta_2_NiSe_5_ and Bi_2_Se_3_. (b) Raman plots corresponding to Ta_2_NiSe_5_, Bi_2_Se_3_ flakes and the heterojunction region. The lower layers are labeled according to their Raman peaks. (c) PL spectra corresponding to Ta_2_NiSe_5_, Bi_2_Se_3_ flakes and the heterojunction region. The laser excitation wavelength is 532 nm and the embedded figure shows a schematic diagram of the PL quenching process. (d) (i) Optical microscopy image of the Ta_2_NiSe_5_/Bi_2_Se_3_ vdWs heterojunction (the gold inter-electrode channel is 8 µm); (ii) SEM electron microscopy (the scale bar is 4 µm) and (iii) EDS spectra of the photodetector. (e) AFM morphology of the heterojunction region and its corresponding thickness measured along the white dashed line. Adapted/reproduced from ref. 72 with permission from Wiley & Sons, copyright 2018.

The Ta_2_NiSe_5_/Bi_2_Se_3_ heterostructure photodetectors reported by Xiao *et al.*^72^ demonstrated self-powered room-temperature operation with responsivity of 0.48 A W^−1^ and specific detectivity of 3.8 × 10^11^ cm Hz^(1/2)^ W^−1^. The response time of 151 µs and polarization ratio of 2.83 enable infrared-coded communication and dual-channel imaging applications. The polarization sensitivity combined with room-temperature self-powered operation addresses requirements for compact multifunctional detectors (Fig. 8).

### 5.4 Application demonstrations

The translation of laboratory photodetector demonstrations to practical imaging and sensing systems represents an essential step toward commercial deployment. Several recent works have progressed beyond single-device characterization to demonstrate array-level performance and system integration capabilities.

Geum *et al.*^73^ demonstrated a 5 × 5 photodetector array based on MoS_2_/In_0.53_Ga_0.47_As heterojunctions exhibiting good uniformity in Raman spectra and clear rectifying characteristics. The devices showed high responsivities corresponding to detectivity of approximately 10^10^ jones at −3 V for incident broadband light from 400 to 1550 nm, with fast photoresponse in the microsecond range for both visible (638 nm) and shortwave infrared (1310 nm) wavelengths. Image scanning demonstrations for visible and infrared light indicate suitability for integration on focal plane arrays.

Liu *et al.* demonstrated a GeSe/PbSe photoinverter with voltage responsivity of 1.26 × 10^6^ V W^−1^ for spectroscopic readout.^74^ The paper also reports a circuit-level D* of 5.8 × 10^14^ jones, but that value is excluded from Fig. 6 and from cross-device rankings because the photoinverter voltage gain and its noise basis are not directly comparable with conventional photodiode or phototransistor D*. The application demonstration is retained without treating the circuit metric as a detector record.

Cai *et al.* reported an IGZO/WSe_2_/PbSe detector with responsivity of 644.4 A W^−1^ and D* on the order of 10^13^ jones at 960 nm, with 56/112 µs rise/fall times.^75^ The WSe_2_ interlayer was used to mediate the interface and the device was demonstrated in a tunable-diode-laser absorption spectroscopy configuration. These data are cited from the dedicated device report rather than from the unrelated source previously assigned to this claim.

### 5.5 Commercial readiness and practical benchmarks

Standalone III–V T2SL detectors and focal-plane products are commercially available from specialist infrared suppliers, demonstrating that the epitaxy, passivation, hybridization to readout circuits, and array calibration can reach product level.^76–78^ No commercial T2SL/2D hybrid product was identified in the surveyed portfolios. Published hybrids remain mainly single or small-area devices, frequently operated at 77 K, and reports of wafer-scale yield, operability, NETD, environmental qualification, and long-term bias stability are scarce.

The practical gate is therefore not another isolated responsivity maximum. A deployable hybrid must provide a reproducible advantage over a standalone T2SL, nBn, CQD, or other control after cooling power, readout compatibility, added transfer steps, contamination risk, pixel pitch, uniformity, and cost are included. Near-term opportunities are likely to be specialised tunable or gain-assisted pixels where the 2D layer supplies a function unavailable from the baseline stack; broad imaging deployment requires wafer-scale integration and array-level reliability data.

## 6. Challenges, controversies, and future directions

### 6.1 Fundamental material limitations

Despite substantial progress, fundamental material constraints continue to limit the performance achievable in type-II heterostructure infrared photodetectors. The narrow bandgaps required for long-wavelength detection inherently enhance thermal carrier generation, establishing a fundamental trade-off between spectral coverage and noise that cannot be overcome through device engineering alone.

Graphene's exceptionally low optical absorption of 2.3% per layer limits photoresponsivity in graphene-based devices despite its superior carrier mobility and broadband absorption capability.^79^ The photogating and gain mechanisms employed to overcome this limitation introduce trade-offs with response speed and linearity that may be acceptable for some applications but problematic for others.^80^ The use of graphene as transparent electrodes rather than primary absorbers, as in the vertical channel devices, represents one approach to leveraging graphene's advantages while circumventing the absorption limitation.^81^

**Fig. 9:**
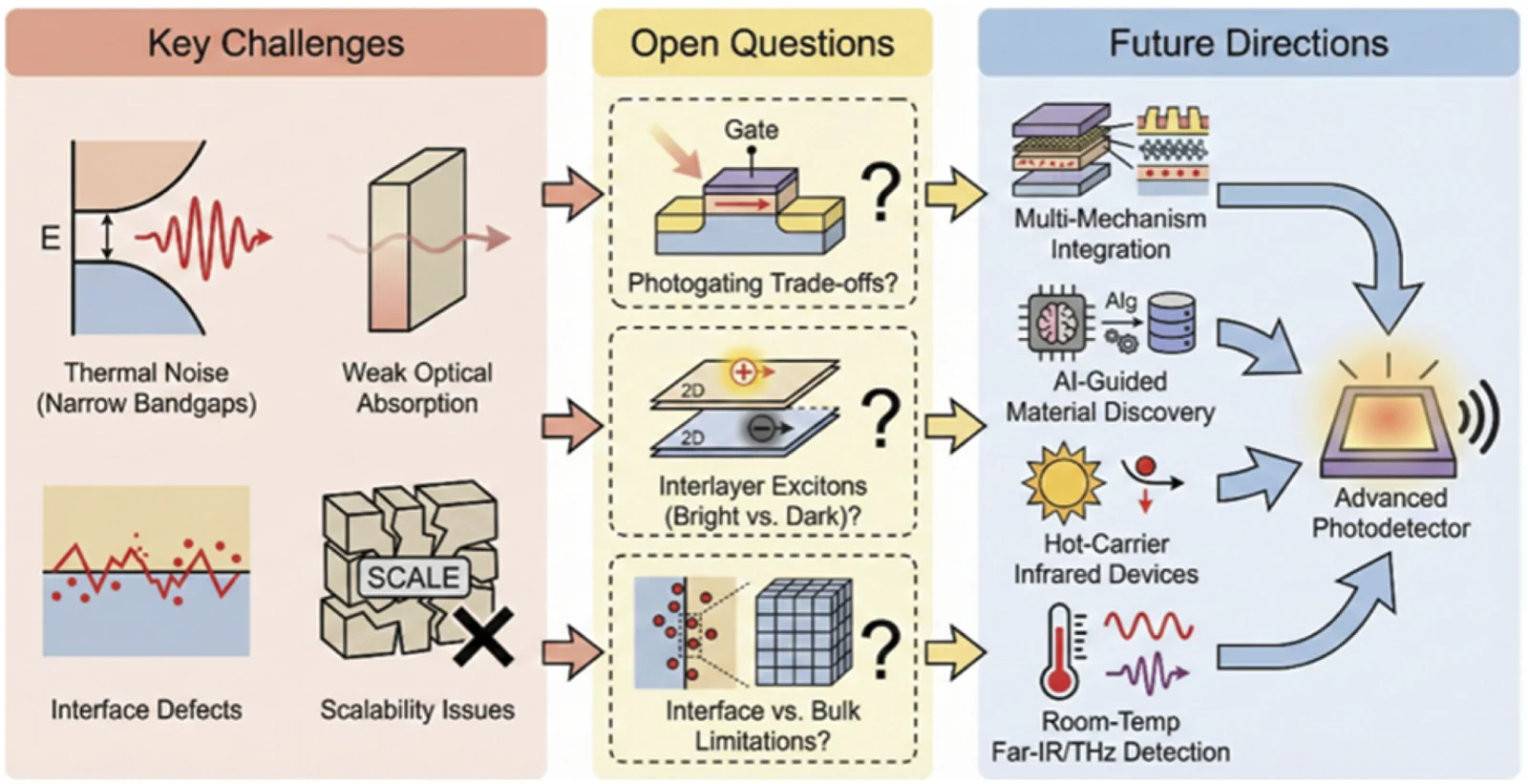
Conceptual overview of the key challenges, debated enhancement mechanisms, and emerging research directions in type-II superlattice/2D nanomaterial infrared photodetectors.

The weak optical absorption in monolayer TMDCs similarly constrains responsivity, motivating the development of enhancement strategies including plasmonic coupling and photogating.^82^ Li *et al.* explicitly noted that the narrow absorption bands and weak optical absorption of MoS_2_ present challenges for near-infrared detection that their InAs@ZnSe core–shell sensitization strategy addresses.^83^ The signal-to-noise ratio improvement of 3–4 orders of magnitude achieved through this approach demonstrates that material limitations can be substantially mitigated through heterostructure design.^84^Fig. 9 provides a conceptual overview summarizing the intertwined material limitations, debated physical mechanisms, and emerging research directions discussed in this section. Rather than focusing on specific device architectures, this schematic highlights the systemic challenges that currently constrain infrared detector performance and outlines potential pathways toward overcoming these bottlenecks.

### 6.2 Manufacturing and scalability challenges

The transition from laboratory demonstrations using mechanically exfoliated materials to scalable manufacturing processes remains a critical barrier for commercial deployment. Chemical vapor deposition (CVD) growth of 2D materials offers a pathway toward large-area production, but achieving the crystalline quality and uniformity of exfoliated flakes has proven challenging.

Martyniuk *et al.*^85^ identified high costs and complex growth techniques for III–V and II–VI materials as persistent manufacturing challenges, with instability and lattice mismatch creating defects that degrade performance. The difficulty of depositing TMDCs on different substrate materials while maintaining quality represents a specific scalability constraint for mixed-dimensional heterostructures. Process development for the wet transfer of 2D materials, as reported by Jiang *et al.*,^86^ addresses some of these concerns but introduces additional complexity relative to monolithic growth.

The device stability, mass production costs, and uniformity issues identified by Wang *et al.*^87^ in their assessment of 2D photodetector prospects underscore the remaining work required before widespread commercialization. Integration into flexible and wearable electronics, while representing an attractive application space, introduces additional fabrication challenges related to substrate compatibility and mechanical robustness.^88^

### 6.3 Theoretical *vs.* experimental performance gaps

Theory–experiment discrepancies arise at several levels. Semilocal DFT underestimates or otherwise misrepresents quasiparticle gaps; GW and hybrid-functional corrections depend on dielectric screening, while optical absorption additionally contains excitonic effects.^28,29^ A freestanding, perfectly registered calculation also omits substrate polarization, encapsulation, finite temperature, strain gradients, twist, reconstruction, contact-induced doping, adsorbates, oxidation, transfer residues, and the distribution of point defects present in a fabricated device. In 2D systems, environmental screening can renormalize both the quasiparticle gap and exciton binding energy, sometimes producing apparent agreement through error cancellation rather than a correct microscopic description.^89,90^

Meaningful validation therefore requires like-for-like observables. Calculated quasiparticle gaps should be compared with photoemission or tunnelling spectra; optical gaps and exciton energies with absorption or photoluminescence; band offsets with photoemission or Kelvin-probe measurements; and device D* only with a transport-and-noise model that includes contacts, traps, bias, area, and temperature. Computational studies should disclose functional, spin–orbit treatment, supercell, registry, vacuum, *k* mesh, basis cutoff, convergence tests, and whether Coulomb truncation and dipole corrections were applied. Uncertainty ranges and alternative interface registries are more informative than a single nominal band diagram.

A practical validation hierarchy is: (i) converge the ideal interface and test registry, strain, and defect sensitivity; (ii) measure the actual composition, morphology, and chemical state; (iii) compare a spectroscopic observable before assigning a device mechanism; and (iv) verify the predicted advantage in a matched device/control pair. This hierarchy prevents a favourable calculated type-II alignment from being treated as proof of low dark current or high detectivity.

### 6.4 Competing perspectives on enhancement mechanisms

The relative contributions of different physical mechanisms to observed performance improvements remain subject to ongoing debate, with implications for optimization strategies and ultimate performance limits. The photogating effect, while widely invoked to explain high responsivities in 2D heterostructure photodetectors, encompasses several distinct microscopic processes including trap-assisted gain, charge puddle formation, and Fermi level modulation that may dominate under different conditions.^91^

The controversy regarding whether interlayer excitons in type-II heterostructures possess sufficient oscillator strength for efficient absorption has been partially resolved through recent theoretical and experimental work demonstrating bright interlayer transitions in appropriately designed systems.^92^ However, the conditions under which interlayer absorption efficiency approaches that of intralayer transitions remain incompletely characterized, limiting the design space for interlayer exciton-based devices.

The role of interface quality *versus* intrinsic material properties in determining dark current and noise characteristics presents another area of ongoing investigation. While interface trap states clearly contribute to excess noise, the relative importance of interface engineering *versus* bulk material optimization varies across different material systems and device architectures. This uncertainty complicates the prioritization of development efforts and may explain inconsistent results across different research groups working with nominally similar material platforms.

### 6.5 Future research directions

Several promising research directions emerge from the analysis of current achievements and remaining limitations. The integration of multiple enhancement mechanisms within single device architectures, while presenting fabrication challenges, could potentially yield multiplicative performance improvements. Combining barrier engineering for dark current suppression with photogating gain enhancement and plasmonic absorption boosting represents an ambitious but potentially rewarding target.

Artificial intelligence-driven material discovery offers an alternative approach to the systematic but computationally expensive first-principles screening of candidate material combinations. Machine learning models trained on existing heterostructure data could accelerate identification of optimal material pairings and structural configurations for specific application requirements.

The development of hot-carrier infrared optoelectronic devices, while still in early stages, provides a fundamentally different approach to overcoming semiconductor bandgap limitations. A complete description of the underlying mechanisms governing hot-carrier generation, transfer, and collection in heterostructures would enable rational design of devices exploiting these ultrafast processes.

Room-temperature operation of far-infrared and terahertz detectors represents a particularly compelling target given the current reliance on cryogenic cooling or thermal detection mechanisms in this spectral range. The theoretical predictions of bright interlayer excitons in C_3_N/C_3_B heterostructures with binding energies suitable for room-temperature stability warrant experimental investigation that could open new applications in security screening, biomedical imaging, and communications.

## 7. Conclusions

This review distinguishes periodic epitaxial III–V T2SLs, 2D/2D type-II junctions, and true mixed-dimensional T2SL/2D hybrids. The broader 2D literature supplies transferable principles for band alignment, trapping, photogating, and interface chemistry, but only a small subset of studies physically integrates a 2D layer with an epitaxial T2SL.

The strongest hybrid evidence comes from matched controls: a MoS_2_ barrier reduced T2SL dark current by about 100-fold at 77 K, and graphene coupling produced a 217-fold LWIR D* enhancement within a single 77 K study.^6,8^ These results show that 2D layers can function as carrier-selective barriers or photogates; they do not yet establish universal superiority over optimized standalone T2SL arrays.

Performance claims must preserve their conditions. D*, NEP, responsivity, EQE, response time, BLIP, and NETD describe different aspects of a detector, and gain-assisted EQE above 100% necessarily carries implications for noise, linearity, and bandwidth. Cross-platform comparisons require wavelength, temperature, bias, area, bandwidth, and measured noise, with matched controls preferred over record-value tables.

Chemical passivation, bonding control, barrier design, vertical transport, photogating, and optical resonators act on different links in the performance chain. Their combination is promising only when interface treatment, optical absorption, carrier lifetime, noise spectrum, and speed are measured together; otherwise, an apparent gain in one metric may conceal a penalty in another.

Commercially supplied standalone T2SL detector technologies coexist with HgCdTe, QWIP/QDIP, nBn, and emerging colloidal-quantum-dot platforms. In contrast, no commercial T2SL/2D hybrid product was identified in the surveyed vendor portfolios.^76–78^ Hybrid devices remain predominantly small-area laboratory demonstrations, commonly at 77 K. The decisive next steps are wafer-scale interface uniformity, environmental and bias-stress reliability, passivation compatible with readout processing, reproducible noise spectra, focal-plane-array yield, NETD, operability, and cost.

Accordingly, T2SL/2D integration is best viewed as a targeted interface-engineering route rather than a replacement for every infrared-detector platform. Progress will be demonstrated by reproducible array-level advantages under common test protocols and by stable manufacturing flows, not by isolated maxima assembled from incomparable conditions.

## Author contributions

Y. Z. conceptualized the review, designed the literature synthesis framework, and wrote the original draft. W. S. performed literature investigation, data curation, and formal analysis. W. Z. contributed to visualization, comparative interpretation, and manuscript revision. Y. Z. supervised the work and handled project administration. All authors reviewed, edited, read, and agreed to the published version of the manuscript.

## Conflicts of interest

There are no conflicts to declare.

## Data Availability

No primary research results, software or code have been included and no new data were generated or analysed as part of this review.
